# Artificial Neural Network-Based Conveying Object Measurement Automation System Using Distance Sensor

**DOI:** 10.3390/s26020455

**Published:** 2026-01-09

**Authors:** Hyo Beom Heo, Seung Hwan Park

**Affiliations:** Department of Mechanical Engineering, Chungnam National University, 99 Daehak-ro, Yuseong-gu, Daejeon 34134, Republic of Korea; sisgyqja@o.cnu.ac.kr

**Keywords:** geometry measurement, linear spline regression (LSR), artificial neural network (ANN), entry-level distance sensor

## Abstract

**Highlights:**

**What is the main finding?**

- An entry-level sensor combined with moving-window features and an ANN can achieve accurate and stable geometry measurements on a conveyor, despite limitations in sensor resolution and beam characteristics.

**What are the implications of the main findings?**

- The results indicate that entry-level sensors, when paired with appropriate feature engineering and machine learning, can serve as a practical alternative to expensive 3D scanners for inline logistics inspection and box-dimension measurement.
- The identified limitations highlight the need for future models that explicitly account for variations in the conveyor speed, sampling rate, and distance between the sensor and object.

**Abstract:**

Measuring technology is used in various ways in the logistics industry for defect inspection and loading optimization. Recently, in the context of the fourth industrial revolution, research has focused on measurement automation combining AI, IoT technologies, and measuring equipment. The 3D scanner used for field logistics measurements offers high performance and can handle large volumes quickly; however, its high unit price limits adoption across all lines. Entry-level sensors are challenging to use due to measurement reliability issues: their performance varies with changes in object location, shape, and logistics environment. To bridge this gap, this study proposes a systematic framework for geometry measurement that enables reliable length and width estimation using only a single entry-level distance sensor. We design and build a conveyor-belt-based data acquisition setup that emulates realistic logistics transfer scenarios and systematically varies transfer conditions to capture representative measurement disturbances. Based on the collected data, we perform robust feature extraction tailored to noisy, condition-dependent signals and train an artificial neural network to map sensor observations to geometric dimensions. We then verified the model’s performance in measuring object length and width using test data. The experimental results show that the proposed method provides reliable measurement results even under varying transfer conditions.

## 1. Introduction

Geometry measurement refers to quantifying the geometric shape and dimensions of an object using measuring instruments, and it is a fundamental technology for facility automation in various industrial fields such as palletizing and de-palletizing [1,2,3], mobile autonomous systems [4,5,6,7], and quality control [8,9,10]. In the logistics domain, this capability underpins smart logistics, where advanced information technology (IT) and intelligent systems are incorporated into the overall process of transport, storage, unloading, packaging, and warehouse operations. As logistics companies increasingly adopt smart logistics platforms to reduce costs and manage processes efficiently, logistics infrastructure is shifting from labor-intensive systems to automated systems, and the importance of reliable logistics measurement for defect inspection and loading optimization continues to grow. Measurement technologies are already widely used in manufacturing and line processes as well as in logistics processes.

There are two broad categories of geometry measurement methods: active systems and passive systems. Active systems rely on optical professional equipment such as three-dimensional (3D) scanners and structured illumination devices, enabling geometry measurement from sensor output without additional processing. These systems provide high measurement performance and are effective for 3D reconstruction, but they are expensive [11] and have limitations in real-time measurement of moving objects in practical environments [8]. Passive systems, in contrast, typically use multi-vision configurations that mimic human binocular or multi-ocular vision to infer 3D information from images [12,13,14,15,16,17]. Mustafah et al. [13] measured the geometry by estimating parameters based on the object’s center point difference between two images captured through a stereo vision system. Ma et al. [15] used a trinocular vision system to obtain higher measurement performance and reliability than the binocular vision system. Zhou et al. [17] proposed calibration, object detection, and geometry measurement methods to measure underwater objects and showed high measurement performance for various objects. Nevertheless, passive systems are sensitive to variations in illumination, object texture, and viewpoint, which can cause object corners or boundaries to appear unclear and degrade measurement performance.

To address some of these issues, research has also examined monocular camera-based geometry measurement methods [18,19,20,21,22,23]. Since images taken with a monocular camera do not have depth information, the distance from the camera to the object and the camera’s focal length is needed [24], or a reference object that serves as a standard for geometry measurement is needed [25,26,27]. Pu et al. [19] proposed double shot calibration that can be used when measuring the distance from the camera to the object is limited, and the proposed method can measure objects with more straightforward calibration than the passive system. Ankad et al. [22] detected objects through YOLO and used focal length to measure the geometry in real time through CCTV. Li et al. [23] installed a five-degree-of-freedom (5-DOF) sensor at the endoscope tip to accurately determine anomalies through an endoscope. This research proposed a method of measuring objects in real-time using position and direction of endoscope measured by a 5-DOF sensor. It showed high measurement performance despite being an endoscope with low resolution. Othman et al. [20] detected objects using canny edge detection and measured the geometry of the contoured object using a reference object. Although these methods can achieve high measurement performance in certain applications, they typically require complex calibration, object detection, and parameter tuning, which increases computational load and makes robust deployment in real environments challenging.

To overcome the cost and robustness limitations of existing active and passive approaches, entry-level sensor-based 3D measurement has been investigated as a promising alternative. Depending on the configuration of the sensor module, these methods can be categorized into single-sensor and complex multi-sensor configurations. Single-sensor modules provide a simple hardware structure and straightforward data collection for 3D measurement but usually acquire data only from one direction, requiring experimental constraints or additional algorithms to reconstruct full 3D information. Complex sensor modules arrange multiple sensors in spatial configurations to collect data from various directions, thereby compensating for the limitations of a single sensor. For example, multiple position-sensitive detectors (PSDs) [28] or distance sensors can be deployed in an array to enhance measurement coverage and accuracy. However, multi-sensor configurations increase system complexity and cost and can still be sensitive to environmental changes.

At the same time, rapid advances in artificial intelligence (AI) and machine learning have led to active research on learning-based measurement technologies that aim to improve performance and adaptability by learning from data. Deep learning and other machine learning methods have been applied to both sensor-based and vision-based geometry measurement tasks [29,30,31], showing strong potential in extracting robust features and compensating for noise or environmental variability. Nonetheless, there remains a shortage of algorithms that can maintain reliable performance in unlearned or changing environments and under practical constraints such as limited sensor configurations, varying object shapes and positions, and fluctuating logistics conditions. These limitations highlight the need for measurement methods that combine entry-level sensors with data-driven algorithms to achieve reliable and practical geometry measurement in real logistics settings.

In this context, this study focuses on developing a machine learning-based geometry measurement model and experimental methodology that enable reliable measurements using an entry-level distance sensor under various constraints. By constructing a data collection device that simulates real logistics transfer environments and systematically analyzing the acquired sensor data, the proposed approach aims to overcome the reliability limitations of conventional entry-level sensors while avoiding the high cost and complexity of professional 3D scanners and multi-camera systems.

Furthermore, this study proposes a systematic framework that integrates signal processing, robust feature extraction, and machine learning–based modeling to address inherent challenges in measurement reliability arising from sensor noise and environmental variability. In practical logistics scenarios, measurement data are influenced not only by the intrinsic characteristics of the sensor but also by external factors such as object position, geometry, and surrounding conditions. The proposed framework is designed to ensure reliable geometry measurement under noisy and variable conditions, thereby enhancing accuracy and robustness.

The key contribution of this study are as follows.

(1) **Reliable geometry measurement with a single entry-level distance sensor**: We propose a machine learning-based measurement approach that achieves reliable geometry estimation using only one low-cost distance sensor under various transfer conditions, avoiding the cost and integration burden of professional 3D scanners or multi-camera systems.
(2) **Systematic framework for geometry measurement**: We introduce the systematic framework that combines signal processing, robust feature extraction, and machine learning–based modeling to mitigate noise and environmental variability, improving both accuracy and robustness.

## 2. Sign and Motivating Example

### 2.1. Measurement System Design

**Figure 1:**
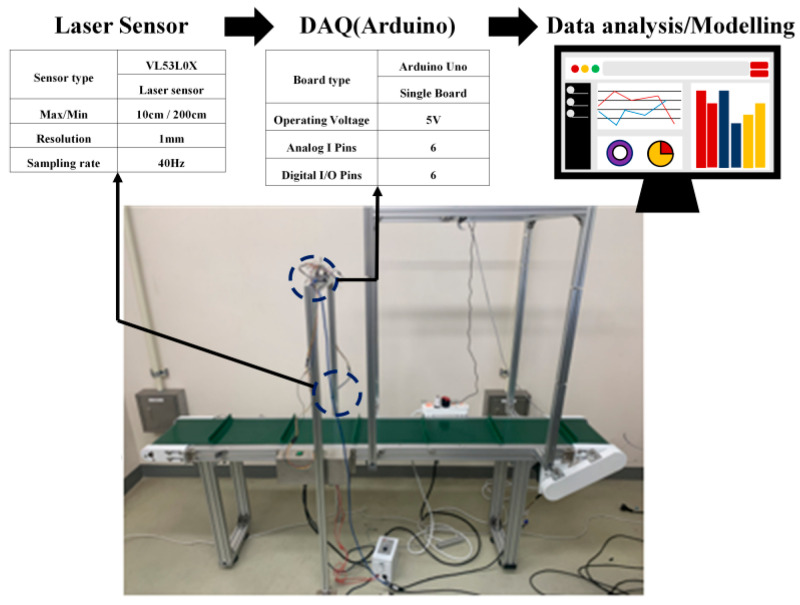
Configuration of a conveyor-based automated measurement system.

A conveyor-based data acquisition system was constructed to emulate an automated measurement environment under uniform experimental conditions. Figure 1 illustrates the configuration of the experimental setup used for measurement automation and data collection. An aluminum profile frame, to which the sensor is mounted, is installed above the center of the conveyor belt, and the sensor acquires data as the belt transports the test object. The side-mounted laser distance sensor measures the object’s length and width, excluding its height. A serial communication interface was implemented, and Python 3.8.2 was used to preprocess and analyze the data transmitted from the Arduino microcontroller. The processed measurement data were then used to develop a measurement model and to monitor the results in the Python environment.

In this study, a VL53L0X time-of-flight (ToF) laser distance sensor [32], a low-cost module compatible with Arduino, was employed to measure objects conveyed by the belt, and its specifications are summarized in Figure 1. The sensor’s operating range of approximately 10–200 cm is sufficient for the dimensions of the experimental conveyor system. In addition, its high resolution and sampling rate provide stable measurements even at relatively high conveyor speeds, enabling experiments under various speed conditions. However, due to the inherent characteristics of ToF sensors, accurate measurement requires that the target objects be composed of bright-colored or highly reflective materials.

Previous work has characterized the measurement performance of the VL53L0X as a function of distance [33]. When the distance between the sensor and a fixed rectangular box was varied, the measurement error increased with distance, but within 50 cm—the maximum sensor-to-object distance used in this study—the standard deviation remained below 0.3 cm, indicating relatively reliable and accurate measurements. Therefore, the VL53L0X sensor was considered suitable for the proposed experimental setup.

An Arduino Uno board was used for data acquisition (DAQ) and sensor control [34]. Arduino is an open-source single-board microcontroller platform that is easy to program and interface with external sensors. Among the available Arduino boards (e.g., Mega, Pro, and Uno), higher-end models with many analog and digital I/O pins were unnecessary because only a single distance sensor was used. Consequently, a lightweight Arduino Uno board was selected as a cost-effective DAQ solution. The bill of materials (BOM) for the proposed conveyor-based data acquisition system is summarized in Table 1. While industrial 3D scanners typically cost on the order of USD 25,000–100,000+ [35], the hardware cost of our proposed system is approximately USD 665, resulting in a substantial reduction in system cost.

The collected distance data were transmitted to a host computer running Python for analysis and modeling. By processing the distance measurements, a system for estimating the length and width of the objects on the conveyor was implemented, and its measurement results were continuously monitored.

### 2.2. Data Description

**Figure 2:**
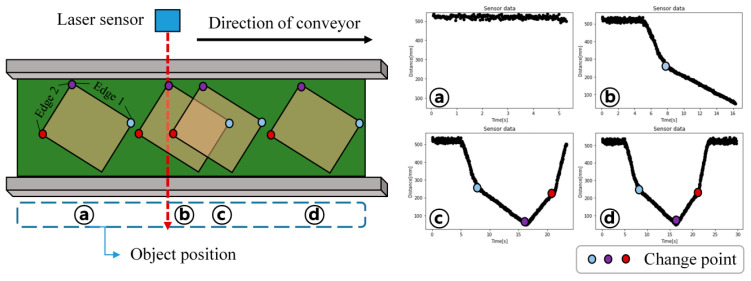
Schematic of the conveyor-based measurement setup and corresponding measured distance signal profiles at different object positions (**a**–**d**), with detected change points indicated.

In this study, the automated measurement system was used to acquire sensor data for object measurement as the object was transported on the conveyor belt. The measurement target was a rectangular box with dimensions similar to those of a standard logistics box. Considering the characteristics of the ToF sensor, a white box with high reflectivity is selected as the target. Although the object height can be easily obtained by mounting an additional sensor on the top of the frame, this study focuses only on estimating the object’s length and width. Figure 2 shows the change in the measured distance signal profile as the object passes through the sensing region on the conveyor. The experiment proceeds from state **a**, before the object enters the sensor field of view, to state **d**, when the object has completely passed and the experiment is finished. Because the laser distance sensor measures the distance from the sensor to the object, a signal profile corresponding to the object’s outline is obtained once the transfer is completed. The object’s vertices correspond to change points in the sensor signal, indicated by the colored dots in Figure 2. When the object length and width are denoted as Edge 1 and Edge 2, respectively, the data can be segmented into the measurement regions of Edges 1 and 2 by identifying the intervals with the minimum distance values in the signal pattern.

**Figure 3:**
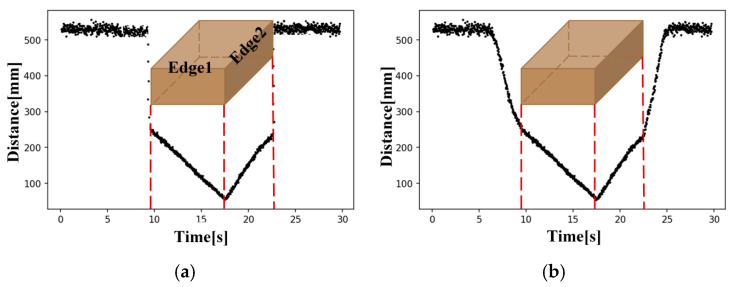
Comparison between (**a**) ideal and (**b**) measured distance signal profiles of the laser distance sensor.

Ideally, the distance signal profile would exhibit a vertical transition when each vertex of the object crosses the sensor’s measurement point, as illustrated by the ideal distance signal profile in Figure 3a. However, in the actual sensor data, these transitions appear as slopes rather than sharp steps. This behavior arises from the measurement characteristics of the sensor used in this study. Industrial high-performance sensors can measure the distance to a moving object with high accuracy owing to their narrow and well-collimated beams. In contrast, the VL53L0X time-of-flight sensor has an emitter and receiver with a conical field of view, so accurate ranging is difficult during the transition period when the object edge enters or leaves the measurement region. As a result, the measured distance gradually changes instead of switching instantaneously. Therefore, an important objective of this study is to minimize the error caused by this sensing characteristic in order to achieve reliable measurement performance.

### 2.3. Motivating Example

This section describes a basic method for estimating the length and width of an object from data collected by the experimental device and discusses its limitations. The most common approach for measuring object length and width with a conveyor-based data collection device is to use the time and distance data at three change points. Chun et al. [36], for example, developed a measurement system that combines an imaging device with two or more sensors to reconstruct the three-dimensional shape of objects transported on a conveyor belt. When an object enters the measurement range, its length and width can be computed from the time and distance data associated with the vertices of the object, as measured by a laser range finder.

To implement measurement automation with the proposed data collection device, an algorithm is required to automatically detect change points corresponding to object edges in the conveyor-belt time-series data. Numerous techniques have been proposed for change-point detection. A basic approach is the moving-window method, in which a window of predefined size is shifted along the time series, and statistics such as the mean or variance of the samples within the window are monitored to identify abrupt changes. This method has low computational complexity and can detect change points quickly, but its performance depends strongly on the choice of window size and detection thresholds. The cumulative sum (CUSUM) chart, widely used in statistical process control, tracks the cumulative sum of deviations from a target value and can detect relatively small shifts compared with other control schemes. For effective application, however, the upper and lower control limits must be carefully determined. Solow [37] proposed a two-phase regression model based on two linear regression segments to estimate a discontinuity in a time series. In this approach, the statistical significance of the estimated breakpoint is evaluated using an F-test, so the user must explicitly select a significance level [38].

Because the detection performance of these methods depends on user-defined parameters, building a robust change-point detector typically requires designing an objective function and performing optimization under various data conditions, which is time-consuming. In our previous study, we employed linear spline regression (LSR), even at the expense of increased computation time, because it can automatically detect change points without requiring manual parameter tuning [33].

**Figure 4:**
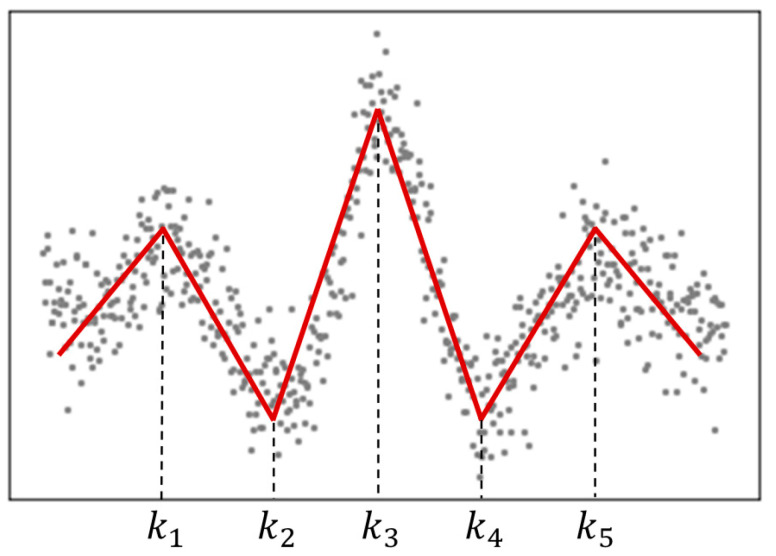
Linear spline regression (*k* = 5).

LSR is a regression-based data-fitting technique that represents nonlinear behavior using multiple linear segments joined at knots, that is, points at which the regression coefficients change. Figure 4 illustrates the result of applying LSR to randomly distributed data. To estimate the optimal regression model, LSR iteratively updates the knot locations, which are initially specified through the user-defined degrees of freedom or number of knots, until the model error is minimized. In general, spline regression can use cross-validation to select the optimal number of knots *k* that yields the most explanatory model. However, because the data in this study consist of piecewise linear patterns, the required number of knots can be determined intuitively from the shape of the observed signal.

**Figure 5:**
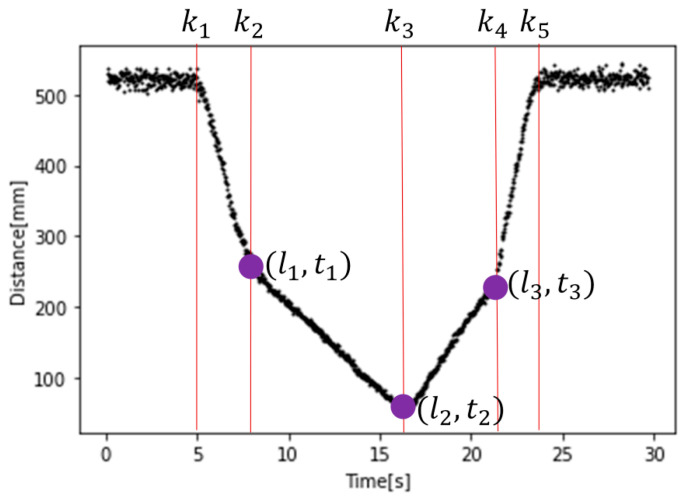
Detecting change point using LSR for object measurement; estimated knots ($k_{1}$–$k_{5}$) and detected change points ($l_{1},t_{1}$), ($l_{2},t_{2}$), ($l_{3},t_{3}$).

Figure 5 shows that the distance signal profile collected by the sensor consist of six linear segments and five change points. Among the five change points detected by applying LSR with *k* = 5, only three ($k_{2},k_{3},k_{4}$) are used for subsequent calculations. The change points corresponding to the object vertices are denoted as ($l_{1},t_{1}$), ($l_{2},t_{2}$) and ($l_{3},t_{3}$). Equations (1) and (2) are then used to compute Edges 1 and 2 from the time and distance information at these three points.

(1) $$Edge1=\sqrt{{{(l}_{1}-l_{2})}^{2}+v^{2}{{(t}_{2}-t_{1})}^{2}},$$

(2) $$Edge2=\sqrt{{{(l}_{2}-l_{3})}^{2}+v^{2}{{(t}_{3}-t_{2})}^{2}},$$

where v denotes the speed of the conveyor belt.

**Figure 6:**
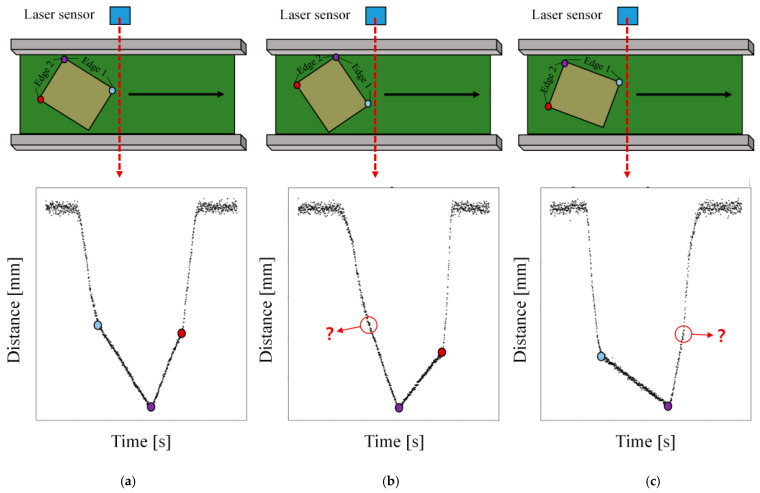
Distance signal profiles under different rotation angle; (**a**) normal case with both change points visible; (**b**) clockwise rotation with an indistinct left change point; (**c**) counterclockwise rotation with an indistinct right change point. The colored dots indicate the object vertices of the conveyed object.

To obtain reliable measurements with the LSR-based geometry measurement algorithm, consistent change point detection performance is required even when transfer conditions such as object size, rotation angle, and sensor sampling rate vary. Figure 6 illustrates the distance signal profiles collected under different rotation conditions of the object. Figure 6a shows a normal case in which all three change points are clearly visible. Figure 6b and Figure 6c present data acquired when the object is rotated clockwise and counterclockwise relative to case (a), respectively, such that the left and right change points become indistinct. Although the three change points in Figure 6a can be detected by LSR and used to measure Edges 1 and 2, the corresponding change points in Figure 6b and Figure 6c cannot be estimated reliably because of degraded detection performance.

## 3. Proposed Model for Geometry Measurement

In summary, when the object transfer conditions change, the geometry measurement performance deteriorates, and a new approach is therefore required. To address this issue, robust features are extracted by analyzing how the distance signal changes under different transfer conditions. In this study, the dynamic pattern of the collected data is used as a feature, and a reliable geometry measurement model is developed based on machine learning.

**Figure 7:**
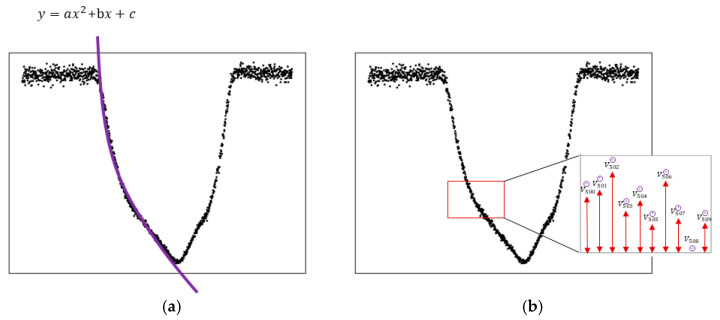
Dynamic pattern extraction techniques from distance signal profiles; (**a**) using data fitting; (**b**) using individual data points.

The data fitting method is used as a representative technique for extracting dynamic patterns. Data fitting represents the signal with a continuous function for interpretation and analysis, and patterns can be extracted from the coefficients of the regression model, as illustrated in Figure 7a. This approach has the advantage of describing the data pattern with a small number of variables, which enables fast computation when used with machine learning, but it yields relatively low measurement accuracy. This is because the collected data consist of piecewise linear patterns, so a nonlinear model obtained by polynomial regression introduces large approximation errors, which directly degrade measurement performance when the transfer conditions change.

Another technique for extracting dynamic patterns is to use individual data points, as shown in Figure 7b. In this approach, each time sample is treated as a separate feature, and the distance value at each time instant is used as the feature’s value. Although this method provides high-resolution information and thus yields accurate measurements, it suffers from high computational cost. In our experiments, one run produces 1250 samples, and a machine learning model that takes 1250 input variables is too complex for real-time measurement. In addition, when the transfer conditions change, the number of input samples can vary, requiring extra preprocessing to align the feature dimension.

For these reasons, this study adopts a moving window-based feature extraction technique, which is widely used in time-series analysis [39,40]. Its basic principle is similar to Figure 7b. However, instead of using every raw data point, summary statistics such as the mean value within each window are used as features.

**Figure 8:**
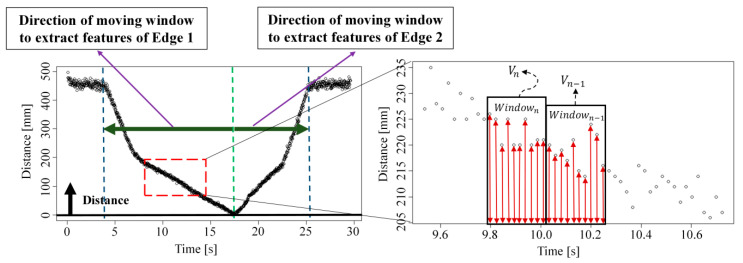
Moving window-based feature extraction for measuring Edges 1 and 2.

Figure 8 illustrates the moving window-based feature extraction technique. Because the distance sensor can produce different absolute values depending on the object position even when the object size is the same, a standardized distance representation is required. Therefore, the distance at each time sample is normalized by shifting the minimum distance value to zero. To extract features for estimating Edges 1 and 2, the moving windows are swept outward in both directions starting from the point with the minimum distance. In addition, to keep the input dimensionality fixed, the window size is chosen such that the number of input variables is 45. The average distance within each window is then computed using Equation (3), where $\bar{d_{n}}$ denotes the mean distance, $d_{i}$ is the distance at the $i^{th}$ sample in the window, and $I_{n}$ is the index of the last sample included in the $n^{th}$ window.

(3) $$\bar{d_{n}}=\frac{\sum_{i=I_{n-1}+1}^{I_{n}}d_{i}}{I_{n}-I_{n-1}}$$

In this study, machine learning is employed to obtain reliable measurements from the dynamic patterns extracted by the proposed feature extraction technique. Machine learning, a subfield of artificial intelligence, enables computers to learn from data in order to perform prediction and classification tasks. By learning the relationship between the extracted features and the ground truth object geometry, a measurement model can be constructed that accurately estimates Edges 1 and 2 even for previously unseen data.

**Figure 9:**
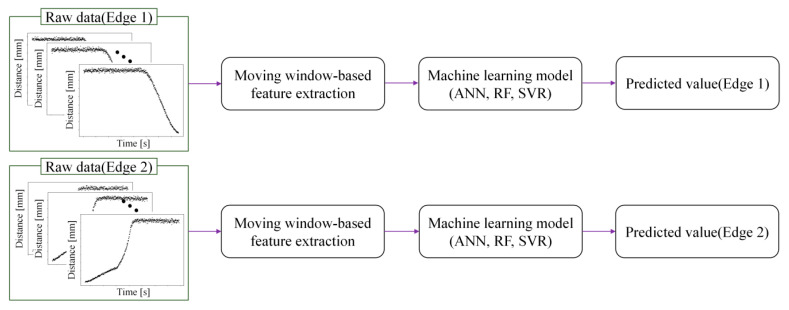
Moving window-based machine learning framework for predicting Edges 1 and 2.

Artificial neural networks (ANNs) are used as the primary machine learning models in this work. ANN are machine learning models inspired by the structure of biological neurons. They consist of an input layer, one or more hidden layers, and an output layer, each composed of interconnected nodes. The nodes are connected by weights that represent connection strengths, and the weights and biases are iteratively updated using error backpropagation to minimize the prediction error. To evaluate the performance of the ANN-based measurement model, its results are compared with those of random forest (RF) and support vector regression (SVR), which are widely used nonlinear regression methods. Figure 9 illustrates the proposed approach for predicting Edges 1 and 2. The method consists of two independent models, one for each edge. First, the distance signal collected by the laser distance sensor is split into raw data segments corresponding to Edge 1 and Edge 2 based on the minimum distance point. Features are then extracted from each segment using the moving window procedure and are input to the respective machine learning model to obtain the predicted edge values.

## 4. Experiments

### 4.1. Design

**Figure 10:**
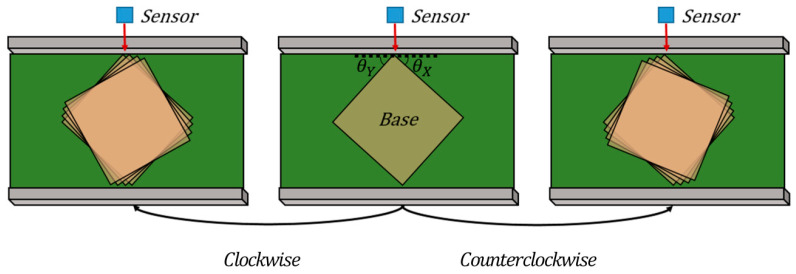
Definition of nine object rotation angles around the base angle.

Table 2 summarizes the experiment scenario used to apply the geometry measurement algorithm proposed in this study. The experiments were conducted using a rectangular box as the test object under a fixed v (27 mm/s), with the experimental scenarios designed by varying only the object size and rotation angle among the possible transfer conditions. Considering the size of the conveyor belt, three standard postal boxes were selected: Type No. 1 (220 mm × 191 mm), Type No. 2 (270 mm × 180 mm), and Type No. 2-1 (350 mm × 251 mm). To reflect diverse transfer conditions, nine rotation angles were defined, as shown in Figure 10. Starting from a base angle at which all change points are clearly detected, the object was rotated in equal angular steps to the clockwise and to the counterclockwise. In total, 27 experimental cases were tested, and each condition was repeated 10 times to collect sufficient data. This study marked on a plate the position of the object corresponding to nine rotation angles. By placing the object at the position corresponding to each rotation angle on the plate and transporting it via a conveyor belt, we ensured consistent rotation angles across repeated experiments. In this process, the rotation angles were measured using a protractor.

The goodness of fit, or learning accuracy, is important for machine learning models, but it is equally crucial to evaluate generalization performance, which indicates how well a model works on unseen data. To assess the generalization performance of the geometry measurement model based on moving window features, experiments were designed in which the model was trained only on specific subsets of the data. Table 3 presents the experimental design, consisting of different combinations of training and test datasets.

Experiments 1 and 2 evaluate whether the model can maintain good measurement performance for data rotated in the opposite direction when it is trained using only clockwise or only counterclockwise rotation angles relative to the base angle. Experiments 3 and 4 assess whether a model trained on central rotation angles can accurately predict measurements at extreme rotation angles, and vice versa. Experiments 5 and 6 examine whether the model trained on intermediate rotation angles can generalize to the remaining unseen angles.

### 4.2. Results

As performance indicators, the mean absolute error (MAE) was used to assess measurement accuracy, and the standard deviation error (SDE) was used to assess measurement variability, as defined in Equations (4) and (5). In addition, RF and SVR were included for comparison to evaluate the performance of the ANN.

(4) $$MAE=\frac{1}{N}\sum_{i=1}^{N}\left|x_{i}-\hat{x_{i}}\right|$$

(5) $$SDE=\sqrt{\frac{1}{N-1}\sum_{i=1}^{N}{\left\{\left(x_{i}-\hat{x_{i}}\right)-mean\;error\right\}}^{2}}$$

Because ANNs are highly sensitive to hyper-parameters, tuning was performed to optimize the number of hidden layers, number of hidden nodes, and learning rate in order to develop an effective deep learning model. Hyper-parameter tuning was also carried out for RF and SVR to ensure a fair comparison of model performance. In this study, grid search implemented in the scikit-learn library was used for hyper-parameter optimization. Table 4 summarizes the tuned hyper-parameters of the three machine learning models. Note that the optimal hyper-parameters differ between Edges 1 and 2 because the geometry measurement models for the two edges were trained independently.

Table 5 presents the prediction performance of the three machine learning models for the five experiments in Table 4. For each experiment, the best-performing model is highlighted in bold. Overall, the ANN achieved the highest measurement performance for Edge 1, whereas RF provided relatively better performance for Edge 2. However, in most cases the ANN exhibited superior accuracy and reliability, indicating that it is a robust model with respect to data variations. Experiments 1 and 2 showed comparatively poor performance for all three models because, in these settings, the training data contain relatively little information relevant to the test data.

In summary, the generalized performance evaluation indicates that the ANN-based geometry measurement model performs best in most experiments, and that performance degrades when the training set does not include data similar to the test conditions, as in Experiments 1 and 2.

**Figure 11:**
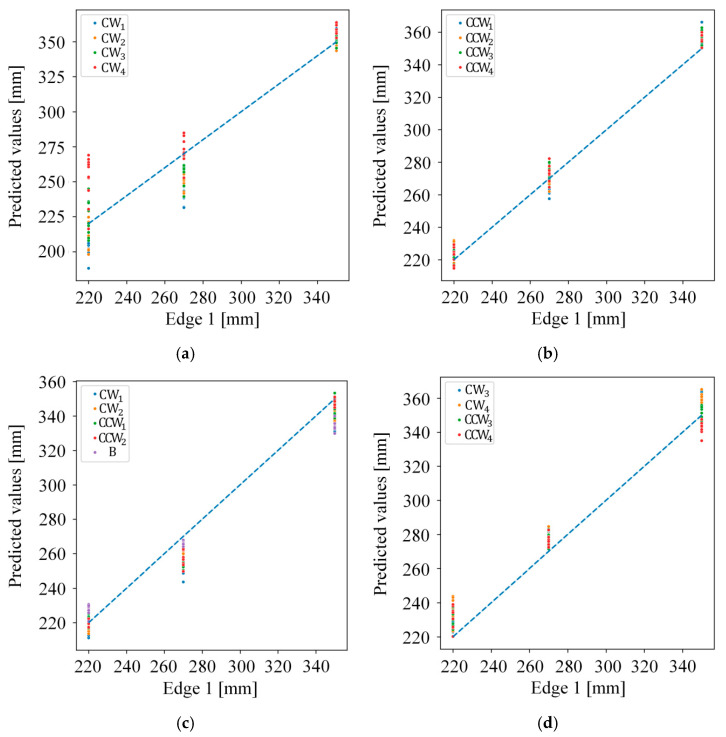

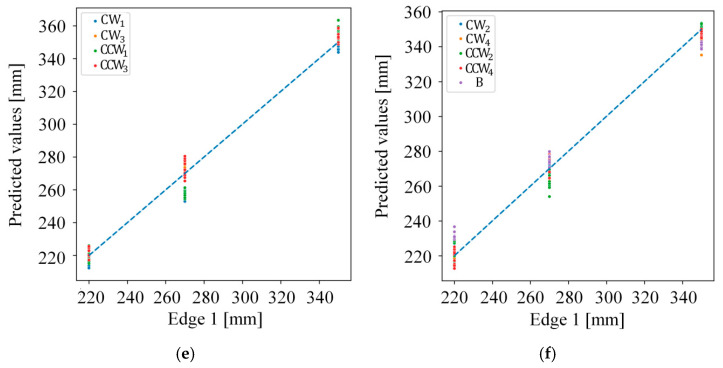
The error analysis of the proposed method for Edge 1 in various experiments; (**a**) Experiment 1; (**b**) Experiment 2; (**c**) Experiment 3; (**d**) Experiment 4; (**e**) Experiment 5; and (**f**) Experiment 6.

**Figure 12:**
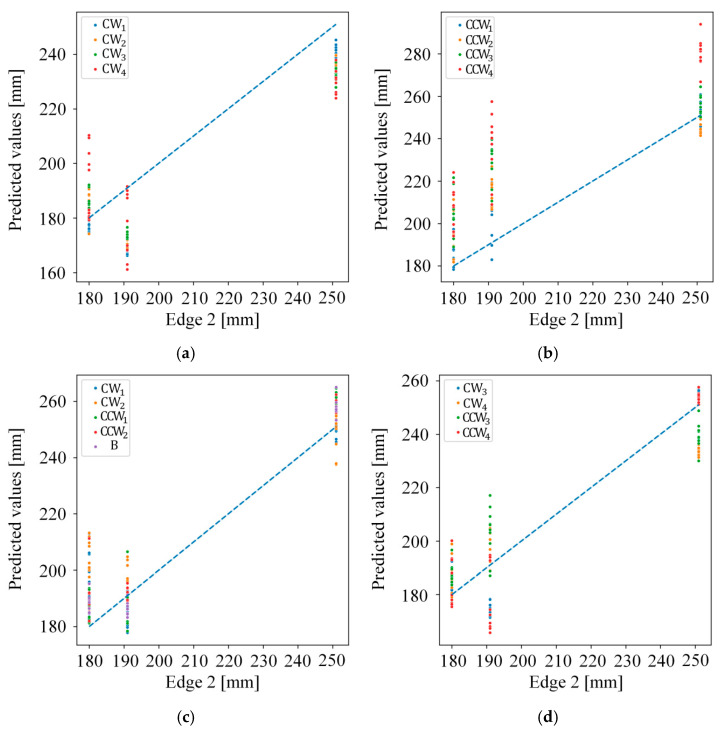

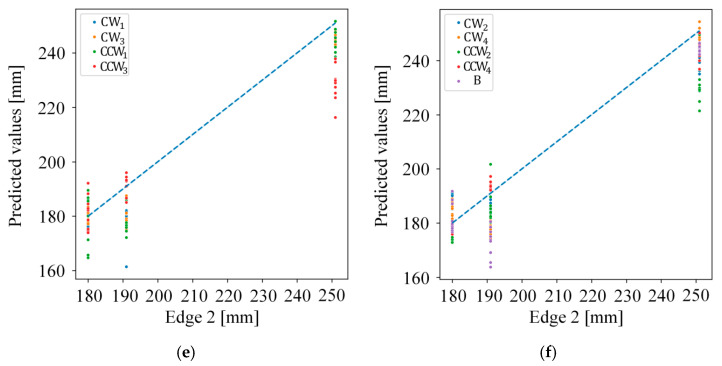
The error analysis of the proposed method for Edge 2 in various experiments; (**a**) Experiment 1; (**b**) Experiment 2; (**c**) Experiment 3; (**d**) Experiment 4; (**e**) Experiment 5; and (**f**) Experiment 6.

Figure 11 and Figure 12 present the results of the error analysis conducted for each experiment using the ANN model that exhibited the best performance. Each point represent the predicted value corresponding to the ground truth value, with the ideal performance indicated by the x = y reference line (blue dashed line). Points close to this line demonstrate high prediction accuracy, while deviations indicate prediction errors.

As indicated in Table 5, Experiments 1 and 2 show higher deviations than the remaining experiments. Although it was anticipated that extrapolation experiments would yield higher errors as the rotation angles differ more from those used during training, the results for Edge 1 in Experiments 2 and 4, which achieved strong performance, indicate a relatively uniform error distribution across rotation angles. By contrast, the results for Edge 1 in Experiment 1 and Edge 2 in Experiments 1, 2, and 4, where performance was relatively limited, followed the anticipated behavior.

**Figure 13:**
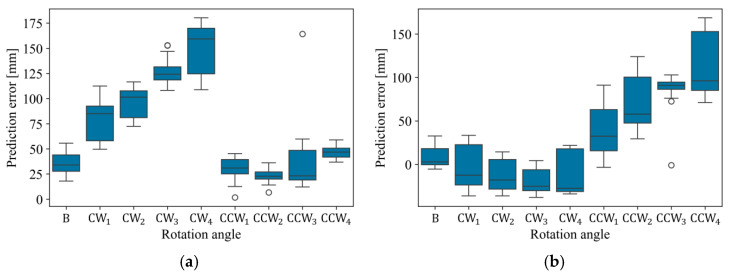
Analysis of LSR Prediction Performance across rotation angles; (**a**) Edge 1 and (**b**) Edge 2. The circles indicate outliers (data points beyond the whiskers).

To compare the performance of the ANN model and LSR, the measurement prediction performance of LSR was evaluated with respect to the rotation angle. Figure 13 illustrates the distribution of prediction errors obtained when LSR was used to predict Edge 1 and Edge 2 based on the data collected under the experimental scenarios described in Table 2. As mentioned in the motivating example, due to the failure to detect knots at certain rotation angles, the prediction performance degrades for Edge 1 under clockwise rotation angles and for Edge 2 under counterclockwise rotation angles. Furthermore, since the ANN model exhibits relatively lower error values compared to LSR, it can be confirmed that the proposed method effectively addresses the limitations of the conventional approach (LSR).

The results of the generalization experiments show that the ANN-based geometry measurement model produces meaningful predictions. However, it is still necessary to examine whether the same performance can be achieved when new data, collected under the same transfer conditions, are applied to the model. Therefore, to verify the reproducibility of the model, additional data were collected according to the experimental scenario in Table 3. The data originally used to build the geometry measurement model are referred to as the original data, and the additional data collected for reproducibility analysis are referred to as the new data.

**Figure 14:**
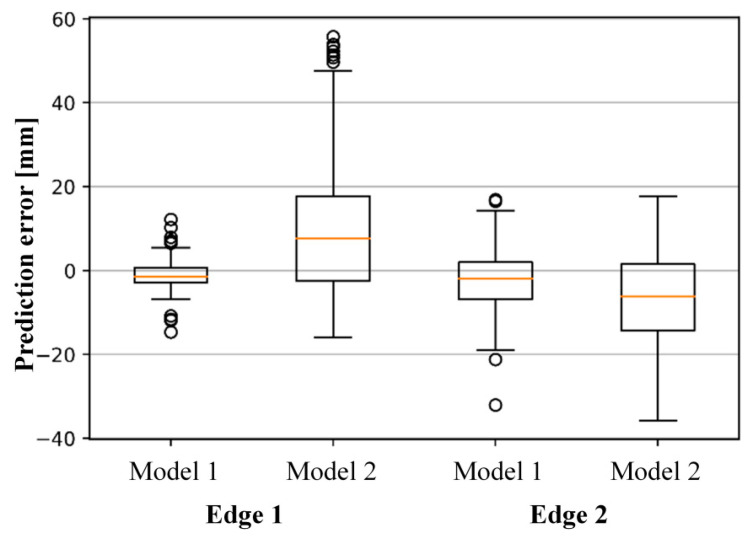
Box plot for reproducibility test results of proposed geometry measurement model.

Reproducibility was evaluated by comparing two experimental settings: Model 1, in which both the training and test sets are generated by evenly splitting the original data under identical transfer conditions, and Model 2, in which the original data are used for training and the new data are used exclusively for testing. The results of the reproducibility experiments for Models 1 and 2 are summarized in Table 6 and Figure 14. For the proposed geometry measurement model to be considered reproducible, the performance of Models 1 and 2 should be similar. However, Table 6 shows that Model 1 outperforms Model 2 for all evaluation metrics, and the interquartile range in the box plots of Figure 14 is much closer to zero for Model 1. These discrepancies indicate that inconsistencies in the process of collecting the new data have led to reduced reproducibility.

**Figure 15:**
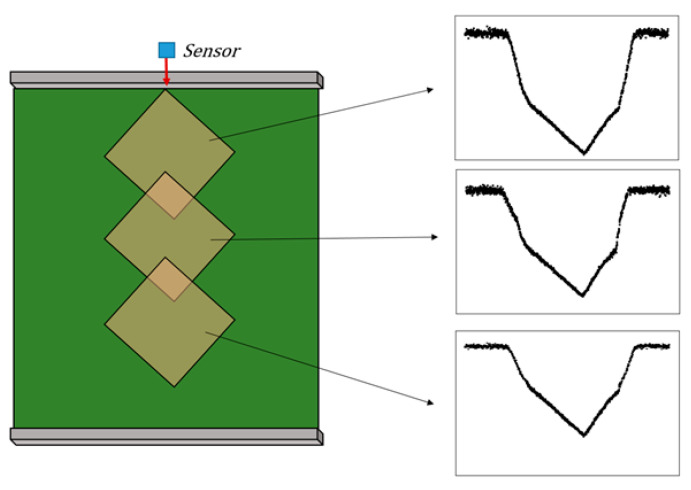
Effect of distance between sensor and object on the distance signal profile.

Figure 15 illustrates how distance signal profile changes as the distance between the sensor and the object varies. Even when the object transfer conditions considered in this study are kept the same, the distance signal profile changes depending on the longitudinal position of the object relative to the sensor. Therefore, to mitigate this issue, it is necessary either to collect sufficient data by placing the object at various positions or to employ deep learning methods to extract meaningful features for geometry measurement directly from the raw data.

## 5. Conclusions

High-performance 3D scanners and imaging-based commercial systems are widely used in logistics facilities. However, their high cost has limited research into more affordable alternatives. Conversely, entry-level sensor-based approaches often suffer from performance degradation under real-world transfer conditions.

This study presented a systematic measurement framework that combines signal processing, robust feature extraction, and machine learning to address the reliability issues caused by sensor noise and environmental variability in logistics measurement. By emulating a conveyor-belt-based logistics environment and collecting data under diverse transfer conditions, the proposed approach demonstrated consistent and reliable geometry measurement performance, even when using entry-level sensors. The results confirm that the integration of these techniques enables accurate and robust measurements despite variations in object position, geometry, and surrounding conditions.

Overall, this study provides a cost-effective alternative for logistics geometry measurement and highlights its potential applicability to broader inline inspection tasks, such as real-time detection of crushed or deformed logistics boxes.

### 5.1. Limitations

Despite the promising results, several limitations of the present study should be acknowledged. The experimental validation was conducted in a controlled laboratory-scale conveyor environment, and the considered transfer conditions were limited mainly to variations in object size and rotation angle. Other operational factors commonly encountered in real logistics facilities, such as changes in the conveyor speed and sensor sampling rate, were not explicitly incorporated into the proposed model.

In addition, a direct performance comparison with a traditional industrial 3D scanner was not performed in this study. Although such a comparison could further strengthen the analysis, conducting a like-for-like evaluation within the proposed conveyor-based measurement setup is practically challenging and may result in an unfair comparison. Industrial 3D scanning systems are typically designed and evaluated under dedicated hardware and software workflows that include scanning, alignment or registration, data fusion, meshing, and post-processing. Integrating these systems into a custom conveyor and instrumentation environment is not straightforward, and their performance is strongly influenced by post-processing procedures that are not compatible with real-time inspection constraints.

Furthermore, based on our preliminary hands-on evaluation using a professional handheld 3D scanner, the complete workflow from data acquisition to software processing and mesh cleaning required in the order of tens of minutes to obtain a fully processed scan under practical conditions. This latency fundamentally differs from the design objective of the proposed approach, which aims at low-latency inline measurement with direct output of the target geometric dimensions. As a result, a direct accuracy-based comparison alone would not adequately reflect the distinct application scenarios and performance criteria of the two approaches.

Finally, the current framework predicts Edge 1 and Edge 2 using separate regression models. While this approach is effective for the present setup, it may limit scalability and increase computational complexity when extending the method to more complex geometry measurement tasks. Furthermore, although robust performance was achieved within the training domain, some degradation in extrapolation capability and reproducibility was observed under conditions that were insufficiently represented in the training data.

### 5.2. Future Work

To enhance applicability in more diverse real-world logistics environments, future research will focus on developing models that explicitly incorporate variations in the conveyor speed and sensor sampling rate. Multi-output regression models that predict Edge 1 and Edge 2 simultaneously within a single machine learning framework will also be investigated to enable immediate geometry estimation in real time.

Furthermore, the deployment of the proposed model on embedded platforms, such as the Raspberry Pi, will be explored to assess real-time performance and practical feasibility in industrial settings. This step is expected to help bridge the gap between laboratory prototypes and real-world deployment.

Finally, to further improve extrapolation performance, reproducibility, and industrial applicability, future work will investigate deep learning-based approaches, such as convolutional neural networks (CNNs), that can directly learn robust representations from raw distance signals acquired under a wider range of longitudinal object positions and box sizes. Training on more diverse transfer conditions and object geometries is expected to enhance measurement stability and address the limitations identified in this study.

## Figures and Tables

**Table 1:** Bill of materials for the proposed conveyor-based data acquisition system.

Main Components	Quantity	Price (USD)
VL53L0X	1	23
Arduino Uno	1	27
Conveyor belt	1	615

**Table 2:** Experimental scenarios for geometry measurement with different object sizes and rotation angles.

	$\theta_{X}$(Degree)	$\theta_{Y}$(Degree)	Size			Iteration
			No.	Edge 1 (mm)	Edge 2 (mm)	
${CCW}_{4}$	16.71	73.29	1	220	191	10 times per size
			2	270	180	
			2-1	350	251	
${CCW}_{3}$	21.71	68.29	1	220	191	10 times per size
			2	270	180	
			2-1	350	251	
${CCW}_{2}$	26.71	63.29	1	220	191	10 times per size
			2	270	180	
			2-1	350	251	
${CCW}_{1}$	31.71	58.29	1	220	191	10 times per size
			2	270	180	
			2-1	350	251	
Base (B)	36.71	53.29	1	220	191	10 times per size
			2	270	180	
			2-1	350	251	
${CW}_{1}$	41.71	48.29	1	220	191	10 times per size
			2	270	180	
			2-1	350	251	
${CW}_{2}$	46.71	43.29	1	220	191	10 times per size
			2	270	180	
			2-1	350	251	
${CW}_{3}$	51.71	38.29	1	220	191	10 times per size
			2	270	180	
			2-1	350	251	
${CW}_{4}$	56.71	33.29	1	220	191	10 times per size
			2	270	180	
			2-1	350	251	

**Table 3:** Experimental design for evaluating generalization performance across different rotation angles.

Experiments	Training Datasets	Test Datasets
Experiment 1	${CCW}_{1},{CCW}_{2},CCW_{3},CCW_{4},B$	${CW}_{1},CW_{2},CW_{3},CW_{4}$
Experiment 2	${CW}_{1},CW_{2},CW_{3},CW_{4},B$	${CCW}_{1},{CCW}_{2},CCW_{3},CCW_{4}$
Experiment 3	$CCW_{3},CCW_{4},CW_{3},CW_{4}$	${CCW}_{1},{CCW}_{2},B,{CW}_{1},CW_{2}$
Experiment 4	${CCW}_{1},{CCW}_{2},B,{CW}_{1},CW_{2}$	$CCW_{3},CCW_{4},CW_{3},CW_{4}$
Experiment 5	$CCW_{2},CCW_{4},B,CW_{2},CW_{4}$	$CCW_{1},CCW_{3},\;CW_{1},CW_{3}$
Experiment 6	$CCW_{1},CCW_{3},\;CW_{1},CW_{3}$	$CCW_{2},CCW_{4},B,CW_{2},CW_{4}$

**Table 4:** Results of hyper-parameter tuning.

Model	Hyper-Parameter	Value		Search Range
		Edge 1	Edge 2	
ANN	Hidden layers	4	4	[1~10], step = +1
	Hidden nodes	160	20	[20~140], step = +20
	Learning rate	0.01	0.01	[0.00001~0.1], step = ×10
RF	Estimators	50	25	[25~150], step = +25
	Max features	8	2	[2~10], step = +2
SVR	Kernel	Linear	RBF	[Linear, Poly, RBF]
	Cost	0.1	100	[0.001~100], step = ×10

**Table 5:** Comparison of prediction performance of machine learning models.

Experiments	Model	Edge 1 [mm]		Edge 2 [mm]	
		MAE	SDE	MAE	SDE
Experiment 1	ANN	**12.72**	17.14	**13.86**	**12.68**
	RF	15.21	23.79	16.91	21.25
	SVR	13.72	**17.08**	19.78	19.04
Experiment 2	ANN	**5.83**	**5.76**	20.28	**17.39**
	RF	37.82	34.86	16.47	19.42
	SVR	16.90	17.86	**15.85**	16.09
Experiment 3	ANN	**9.00**	**8.56**	8.10	9.39
	RF	16.14	15.97	**6.28**	**7.14**
	SVR	9.23	10.67	8.50	9.87
Experiment 4	ANN	**9.08**	**7.04**	9.62	11.81
	RF	22.95	28.27	11.80	16.17
	SVR	16.60	21.19	**8.47**	**11.73**
Experiment 5	ANN	**5.06**	**6.58**	8.36	8.22
	RF	10.22	14.79	**4.84**	**5.99**
	SVR	9.72	11.59	7.24	9.94
Experiment 6	ANN	**5.38**	**6.47**	7.60	**8.21**
	RF	13.93	21.28	7.71	12.98
	SVR	8.84	10.59	**6.93**	9.87

Values in bold indicate the best performance across methods.

**Table 6:** Reproducibility test results of proposed geometry measurement model.

Model	Edge 1 [mm]		Edge 2 [mm]	
	MAE	SDE	MAE	SDE
Model 1	2.88	2.57	6.03	5.24
Model 2	15.84	15.41	11.46	8.12

## Data Availability

We uploaded the dataset we used for this study. Please refer to the following link. Download link: https://github.com/midalab/Geometry-measurement/ (accessed on 5 January 2026).

## Abbreviations

The following abbreviations are used in this manuscript:

AI	Artificial intelligence
IoT	Internet of things
DOF	Degree of freedom
LSR	Linear spline regression
ANNs	Artificial neural networks
IT	Information technology
PSDs	Position sensitive detectors
ToF	Time of flight
DAQ	Data acquisition
CUSUM	Cumulative sum
SVR	Support vector regression
RF	Random forest
MAE	Mean absolute error
SDE	Standard deviation error
CNNs	Convolutional neural networks
